# Biogenic ZnO Nanoparticles Synthesized Using a Novel Plant Extract: Application to Enhance Physiological and Biochemical Traits in Maize

**DOI:** 10.3390/nano11051270

**Published:** 2021-05-12

**Authors:** Daniele Del Buono, Alessandro Di Michele, Ferdinando Costantino, Marco Trevisan, Luigi Lucini

**Affiliations:** 1 Dipartimento di Scienze Agrarie, Alimentari e Ambientali, University of Perugia, Borgo XX Giugno, 06121 Perugia, Italy; daniele.delbuono@unipg.it; 2 Department of Physics and Geology, University of Perugia, via Elce di Sotto, 06123 Perugia, Italy; alessandro.dimichele@collaboratori.unipg.it; 3 Dipartimento di Chimica, Biologia e Biotecnologia, University of Perugia, Via Elce di Sotto 8, 06123 Perugia, Italy; 4 Department for Sustainable Food Process, Università Cattolica del Sacro Cuore, 29122 Piacenza, Italy; marco.trevisan@unicatt.it (M.T.); luigi.lucini@unicatt.it (L.L.)

**Keywords:** biogenic nanoparticles, duckweed, plant stimulation, phytochemicals, antioxidant activity

## Abstract

The need to increase crop productivity and resistance directs interest in nanotechnology. Indeed, biogenic metal oxide nanoparticles can promote beneficial effects in plants, while their synthesis avoids the environmental impacts of conventional synthetic procedures. In this context, this research aimed to synthesize biogenic zinc oxide nanoparticles (ZnO-NPs) using, for the first time, an extract of a wild and spontaneous aquatic species, *Lemna minor* (duckweed). The effectiveness of this biogenic synthesis was evidenced for comparison with non-biogenic ZnO-NPs (obtained without using the plant extract), which have been synthesized in this research. XRD (X-ray diffraction), FE-SEM (field emission gun electron scanning microscopy), EDX (energy dispersive x-ray spectroscopy), TEM (transmission electron microscope) and UV-vis (ultraviolet-visible spectrophotometry) showed the biogenic approach effectiveness. The duckweed extract was subjected to UHPLC-ESI/QTOF-MS (ultra high-pressure liquid chromatography quadrupole time of flight mass spectrometry) phenolic profiling. This untargeted characterization highlighted a high and chemically diverse content in the duckweed extract of compounds potentially implicated in nanoparticulation. From an application standpoint, the effect of biogenic nanoparticles was investigated on some traits of maize subjected to seed priming with a wide range of biogenic ZnO-NPs concentrations. Inductive effects on the shoot and root biomass development were ascertained concerning the applied dosage. Furthermore, the biogenic ZnO-NPs stimulated the content of chlorophylls, carotenoids, and anthocyanin. Finally, the study of malondialdehyde content (MDA) as a marker of the oxidative status further highlighted the beneficial and positive action of the biogenic ZnO-NPs on maize.

## 1. Introduction

Nanotechnology is attracting increasing interest due to the many possible applications in various scientific areas. Several nanomaterials of different origin can be used for a variety of technological applications [1]. Diverse approaches can be adopted to produce nanomaterials [1]. They range from synthetic procedures that can have a high environmental impact to those based on biological entities, such as plant extracts, that have immediate advantages being much eco-friendlier and more biocompatible [2,3]. Among the various nanomaterials, oxides of certain metals in the range of 1–100 nm attract particular attention for their chemical, mechanical, electronic and biological properties [4,5]. Generally, the properties of nanoparticles (NPs) are closely linked to their size and shape, which is why the possibility of being able to control these characteristics plays a significant role in conferring the desired attributes to the resulting nanomaterials [6,7].

NPs synthesis can be carried out using some plant extracts as reducing, ligation and capping agents, thanks to the considerable content of these biological materials of certain classes of compounds [8,9]. Indeed, these substances have a catalytic impact on the formation of NPs and influence their shape, size and morphology [10,11]. In this regard, the literature reports that certain plant extracts can be used to synthesize metal oxide nanoparticles, especially zinc oxide (ZnO-NPs). In particular, ZnO-NPs are safer and lesser toxic than other metal oxides [2]. Recent studies have proposed using ZnO-NPs for their antifungal, antiviral, and antibacterial activity [12]. Furthermore, ZnO-NPs are particularly valuable in developing materials with UV filtering or photochemical activity [13].

NPs have also been used in agriculture as, in small concentrations, they can improve physiological, morphological and biochemical traits in the treated species [4,5,14]. Several studies have shown that NPs can stimulate plant germination, photosynthesis, and development [5,7,14,15]. Moreover, biogenic ZnO-NPs have been shown to increase crop resistance to abiotic stresses such as salinity, drought or toxic metals [16]. Plant extracts efficacy in the biogenic synthesis of ZnO-NPs has been documented on a wide variety of molecules they contain. Plant with high contents of alkaloids, anthocyanins, phenolic acids, proteins, flavones, flavonols, flavanones, phenols and polyphenolic compounds have been found effective in the synthesis of ZnO-NPs [2,8,17,18,19,20,21]. Among the mechanisms proposed by which these biomolecules may operate in NPs formation, hydroxyl groups can form complexes with zinc [10]. Moreover, carbonyl groups have been hypothesized to complex Zn^2+^, thus stabilizing the NPs and preventing excessive growth [18]. Therefore, identifying plant species with the right type and amount of biomolecules allow hypothesizing a green synthetic approach to obtain ZnO-NPs, thus avoiding environmental impacts associated with the conventional synthetic techniques.

Several published studies have demonstrated the effectiveness of extracts obtained from some plants for ZnO-NPs synthesis. To mention a few examples, recently, *Ulva Lactuca*, an alga, has been used to synthesize ZnO-NPs with a size of 18–27 nm and spherical shape [4]. Fowsiya et al. [20] used a medicinal small tree plant, *Carissa edulis*, for the biogenic synthesis of zinc oxide NPs with an average size of 50–55 nm and flower-shaped morphology. Soto-Robles et al. [21] obtained NPs with a hexagonal shape and size depending on the concentration of the extract of *Justicia spicigera*, an evergreen shrub. Álvarez-Chimal et al. [17] used a perennial herbaceous plant to obtain ZnO-NPs of almost spherical shape and dimensions in the range of 5–30 nm. Singh et al. [22] synthesized with an extract of *Aloe barbadensis Mill* ZnO-NPs of 35 nm size in the form of spherical aggregates. All these studies indicated the plants used, and their metabolites substantially impact particles size and shape. However, the research for the biogenic synthesis of metal oxide NPs has generally focused on terrestrial plants or algal extracts. To date, no studies have been done on biomasses from freshwater aquatic plants. Several freshwater plants may be of interest as they are incredibly productive, fast-growing, and adapted to very different and even hostile conditions, indicating high amounts of antioxidants and protective compounds [23].

In this respect, *Lemna minor* (duckweed), a small floating species used in ecological and phytoremediation studies, is of particular interest [24,25,26]. This species, native to several continents, is characterized by rapid growth and the ability to survive adverse environmental and climatic conditions [27]. Indeed, duckweed can develop in wastewater up to 12.4 g m^−2^ day^−1^ dry weight [28]. Therefore, this plant could represent an attractive renewable feedstock for many different applications [28]. Besides, duckweed is highly resistant to organic and inorganic toxic substances [29], thanks to its high content of antioxidants, which are easily inducible in response to abiotic stresses [23,25].

Based on these premises, a study was carried out to obtain biogenic ZnO-NPs from a duckweed extract and test them on plant growth promotion potential on maize. Considering that duckweed is particularly rich in phenolic compounds [30], the study also aimed to profile these molecules to better corroborating their involvement in the green synthesis of ZnO-NPs. To this end, the crystallographic and dimensional traits of biogenic NPs were compared with those realized using the same approach but without the plant extract. The biogenic NPs were then used for priming maize seeds over a wide range of concentrations, and then physiological and biochemical parameters were investigated on maize seedlings to ascertain the NPs effect on the crop.

## 2. Materials and Methods

### 2.1. Materials

Sodium hypochlorite (NaClO, 98%), potassium nitrate (KNO_3_, 99%) calcium nitrate tetrahydrate (Ca(NO_3_)_2_ × 4H_2_O, 98%), magnesium sulphate heptahydrate (MgSO_4_ × 7H_2_O, 98%), potassium sulphate (K_2_SO_4_, 98%), potassium chloride (KCl, 99%), potassium phosphate monobasic (KH_2_PO_4_, 99%), di-potassium hydrogen phosphate (K_2_HPO_4_, 98%), boric acid (H_3_BO_3_, 99.5%), manganese sulphate monohydrate (MnSO_4_ × H_2_O, 98%), copper sulphate (CuSO_4_, 99%), zinc sulphate heptahydrate (ZnSO_4_ × 7H_2_O, >99%), ammonium molybdate tetrahydrate ((NH_4_)_6_Mo_7_O_24_ × 4H_2_O, 99%), ethylenediaminetetraacetic acid ferric sodium salt (Fe-EDTA), trichloroacetic acid (99%) and thiobarbituric acid (>98%) were provided by Sigma-Aldrich (Milan, Italy). Solvents for UHPLC-ESI/QTOF-MS were LCMS grade from Merck-VWR (Milan, Italy). Sodium hydroxide, acetone and ethanol were provided by Carlo Erba (Milan, Italy). All of the chemicals were used as received without additional purification.

### 2.2. Duckweed Growth Conditions

Duckweed was collected from a small freshwater lake located in Perugia (Italy). The harvested plants were disinfected with an aqueous sodium hypochlorite solution (0.5%—*w*/*v*) for 2 min, then rinsed with copious amounts of distilled water, and transferred into polyethylene trays (35 × 28 × 14 cm) containing a nutrient solution composed of 3.46 mmol L^−1^ KNO_3_, 1.25 mmol L^−1^ Ca(NO_3_)_2_ × 4H_2_O, 0.66 mmol L^−1^ KH_2_PO_4_, 0.071 mmol L^−1^ K_2_HPO_4_, 0.41 mmol L^−1^ MgSO_4_ × 7H_2_O, 0.28 mmol L^−1^ K_2_SO_4_, 1.94 μmol L^−1^ H_3_BO_3_, 0.63 μmol L^−1^ ZnSO_4_ × 7H_2_O, 0.18 μmol L^−1^ (NH_4_)_6_Mo_7_O_24_ × 4H_2_O × 2H_2_O, 1 μmol L^−1^ MnSO_4_ × H_2_O, 21.80 μmol L^−1^ FeEDTA, 1 μmol L^−1^ CuSO_4_. These trays were transferred into a growth chamber and submitted to 120 μmol m^−2^ s^−1^ of light intensity (photoperiod: 12/12 h—light/dark) at 24 ± 2 °C. The growth media was renewed every two weeks.

### 2.3. Preparation and Phytochemical Characterization of the Duckweed Extract

Twenty grams of duckweed were washed repeatedly with distilled water and dried in an oven at 50 °C until a constant weight was reached. One gram of dried duckweed was reduced to powder, extracted in 40 mL of a 25% ethanol (*v*/*v*) aqueous solution. The suspension was stirred at 80 °C for 15 min, then centrifuged for 20 min at 8000 rpm and filtered through a 0.22 μm regenerated cellulose syringe membrane into amber glass vials.

Phenolic compounds were profiled according to an untargeted approach through ultra-high-pressure liquid chromatography quadrupole-time-of-flight mass spectrometry (UHPLC-ESI/QTOF-MS Agilent Technologies 6550iFunnel, Santa Clara, CA, USA) as previously reported [31]. Briefly, reverse-phase liquid chromatography separation was done using an Agilent Zorbax Eclipse plus column (50 × 2.1 mm 1.8 μm—Agilent Technologies, Santa Clara, CA, USA) and a linear gradient of acetonitrile (5 to 95% in 34 min) in water. The injection volume was 4 μL, and QTOF-MS acquisition was set in full scan mode (100–1200 m/z, 1 Hz scan rate), and positive polarity and electrospray conditions were optimized in previous experiments [32].

The annotation of phenolics from raw mass features was done in Profinder B.07 (Agilent Technologies, Santa Clara, CA, USA) by combining monoisotopic accurate mass (mass accuracy < 5 ppm) with isotopic spacing and isotopic ratio, following mass and retention time alignment [32,33]. The public database Phenol-Explorer 3.6 (http://phenol-explorer.eu/, accessed on 24 February 2021) was used as the reference in the annotation. Features were annotated only when present within 100% of replications. After that, phenolics were grouped in sub-classes (according to Phenol-Explorer 3.6 annotations), and each cumulative abundance quantified against calibration curves (linear fitting, not forced to origin) prepared from pure standard solutions (Extrasynthèse, Lyon, France), as reported in previous work [31].

### 2.4. Synthesis of ZnO-NPs Using the Plant Extract (Biogenic ZnO-NPs) or Not (Non-Biogenic ZnO-NPs)

To 50 mL of a solution containing 1.40 M ZnSO_4_ × 7H_2_O, 30 mL of the plant extract, obtained as described previously, was added drop by drop. The resulting suspension was kept at 80 °C for 2 h. The solid phase was then recovered by centrifugation, repeatedly washed and centrifuged, and left for 16 h in an oven at 80 °C. After that, the solid was powdered and calcined in a muffle at 400 °C for 4 h. The ZnO-NPs obtained by applying this procedure, based on the use of the duckweed extract, are referred to throughout the text as biogenic ZnO-NPs. The efficiency of the biogenic synthesis was estimated according to Król et al. [34].

The same procedure was used to synthesize zinc oxide NPs without the plant extract, using different volumes. 100 mL of 2 M NaOH were added drop by drop to 50 mL of the solution 1.4 M ZnSO_4_ × 7H_2_O. After the reaction, the suspension was washed and centrifuged repeatedly. The white solid obtained was left for 16 h at 80 °C, then reduced to a fine powder and calcinated in a muffle for 4 h at 400 °C. The ZnO-NPs obtained by applying this procedure, thus without using the duckweed extract, are referred to throughout the text as non-biogenic ZnO-NPs.

### 2.5. XRD, Fe-SEM, TEM and UV-vis Characterization of the ZnO-NPs Obtained

Powder X-Ray Diffraction (PXRD) patterns were collected in reflection geometry in the 10–80° 2θ range, with a 40 s step-1 counting time and with a step size of 0.016° on a PANalytical X’PERT PRO diffractometer (Malvern Panalytical Ltd., Malvern, UK), PW3050 goniometer (Malvern Panalytical Ltd., Malvern, UK), equipped with an X’Celerator detector (Malvern Panalytical Ltd., Malvern, UK) by using the Cu Kα radiation. The long fine focus (LFF) ceramic tube operated at 40 kV and 40 mA. The UV-vis spectra of samples were measured in the range 250–700 nm.

The morphology and composition were examined by field emission gun electron scanning microscopy (FE-SEM) LEO 1525 ZEISS (Zeiss, Jena, Germany). Elemental composition and chemical mapping were determined using a Bruker Quantax EDS (Bruker Nano GmbH, Berlin, Germany). The samples were deposited on conductive carbon adhesive tape and metalized with chromium (8 nm).

TEM images were obtained using a Philips 208 transmission electron microscope (FEI, Hillsboro, OR, USA). The samples were prepared by putting one drop of an ethanol dispersion of the sample powder on a copper grid pre-coated with a Formvar film and dried in air.

### 2.6. Maize Growth Condition and Treatments with Biogenic ZnO-NPs

Maize (cv Belgrano) seeds were surface-sterilized for three minutes with NaClO (0.25%, *v*/*v*). Subsequently, the seeds were rinsed with distilled water. For seed-priming with the biogenic NPs, suspensions containing biogenic ZnO-NPs were prepared at the concentrations of 25 mg L^−1^ (T25), 50 mg L^−1^ (T50), 100 mg L^−1^ (T100) and 200 mg L^−1^ (T200) and sonicated for 5 min. Seeds were immersed in 10 mL of the above solutions for 8 h under slow agitation. Then, the seeds were placed in Petri dishes on paper (10 seeds/plate), added with 10 mL of distilled water, covered and put into a growth chamber in the dark (22 ± 2 °C). Control seeds were obtained for priming with distilled water.

For days after priming and sowing, seedlings were transferred to hydroponic solutions containing a nutrient solution 2 mmol L^−1^ Ca (NO_3_)_2_ × 4H_2_O, 0. 5 mmol L^−1^ MgSO_4_ × 7H_2_O, 0.7 mmol L^−1^ K_2_SO_4_, 0.1 mmol L^−1^ KCl, 0.1 mmol L^−1^ KH_2_PO_4_, 1 μmol L^−1^ H_3_BO_3_, 0.5 μmol L^−1^ MnSO_4_ × H_2_O, 0.5 μmol L^−1^ CuSO_4_, 0.5 μmol L^−1^ ZnSO_4_ × 7H_2_O, 0.01 μmol L^−1^ (NH_4_)_6_Mo_7_O_24_ × 4H_2_O, and 100 μmol L^−1^ Fe-EDTA. Fourteen days after sowing (14 DAS—second leaf stage), plants were harvested and subjected to the following determinations.

### 2.7. Determination of Growth, Photosynthetic Pigments, Carotenoids and Anthocyanins of Maize Plants Treated with Biogenic ZnO-NPs

At 14 DAS (days after sowing), the shoot and root lengths and fresh weight were recorded for samples of maize subjected to the different treatments. Further, the contents of chlorophyll a, chlorophyll b and carotenoids were assessed. In detail, 1.0 g of plant sample was extracted with 85% acetone in water (*v*/*v*). The suspensions were filtered, and the absorbance determined at 452.5, 644, and 663 nm [35].

Maize shoots were harvested at 14 DAS (1 g) and extracted with ethanol (95%) using pestle and mortar to determine the anthocyanin content. The resulting suspension was filtered and centrifuged, and the anthocyanin content was determined spectrophotometrically at 535 and 650 nm [36].

### 2.8. Malondialdehyde Content (MDA) in Treated Maize

The lipid peroxidation was determined in maize seedlings treated with T25, T50, T100 and T200, collected at 14 DAS (days after sowing). Seedlings (0.5 g) were homogenized in a solution containing 10% (*w*/*v*) trichloroacetic acid and 0.25% (*w*/*v*) thiobarbituric acid. The suspensions were then centrifuged at 10,000× *g* for 15 min. The supernatants were heated in a water bath (95 °C). After quick cooling, the MDA content was quantified spectrophotometrically [37].

### 2.9. Statistical Analysis

Each value reported represents the mean of the data from three independent experiments on at least three biological replicates per experiment. Statistical analysis of the data was performed in ANOVA mode by analyzing the variance with Duncan’s test at *p* < 0.05.

## 3. Results

### 3.1. XRD Analysis of ZnO-NPs Synthesized with or without Duckweed Extract

**Figure 1:**
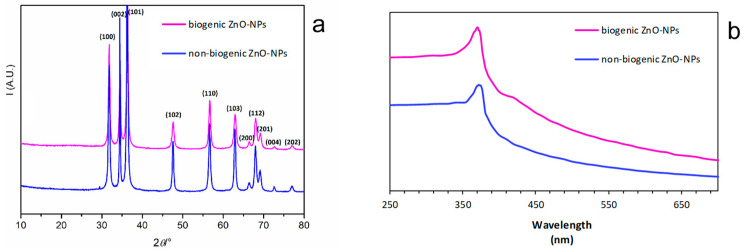
XRPD (**a**) and UV-vis (**b**) patterns of biogenic and non-biogenic ZnO-NPs.

XRPD patterns of ZnO-NPs synthesized with or without the plant extract were investigated (Figure 1a). The biogenic NPs can be indexed as ZnO phase (ICDD n. 00–003-0888). Average crystalline domain size was estimated by applying the Sherrer formula [38] τ = $\frac{K\lambda }{\beta \cos\vartheta }$, with τ = average crystalline domain size and β as integral breadth, on the (1 0 2) isolated peak, after fitting the profile with a pseudo-Voigt function and after subtracting the instrumental contribution by using a profile line reference standard (LaB6). ZnO nanocrystals are in the range 25.5 nm for the non-biogenic NPs and 11.7 nm range for the biogenic ones.

The UV-Vis spectra of the biogenic and non-biogenic ZnO-NPs is reported in Figure 1b. The biogenic ZnO-NPs showed maximum absorption at 369 nm, while the non-biogenic ZnO-NPs showed it at 372 nm. The bandgap energy (Eg) was calculated as Eg = 1240/λ [22], where λ is the maximum absorption wavelength. Eg values were 3.33 eV and 3.35 eV for the biogenic and non-biogenic ZnO-NPs, respectively.

### 3.2. FE-SEM, EDX and TEM Analyses of ZnO-NPs Synthesized with or without Duckweed Extract

**Figure 2:**
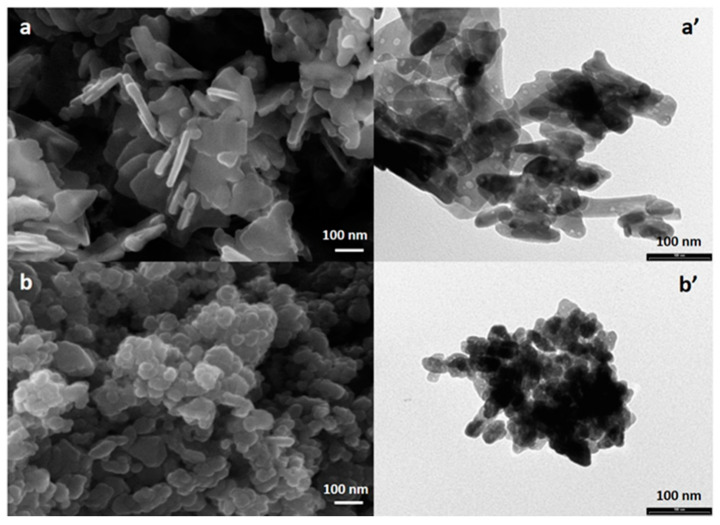
FE-SEM and TEM images of samples non-biogenic (**a**,**a’**) and biogenic (**b**,**b’**) ZnO-NPs.

SEM and TEM images of the non-biogenic ZnO-NPs (Figure 2a,a’) show a nano-lamellar structure with dimension comprised into 20–100 nm range, measured along the lamellae. Biogenic ZnO-NPs (Figure 2b,b’) show a nano-spherical structure with dimensions between 10 and 20 nm.

**Figure 3:**
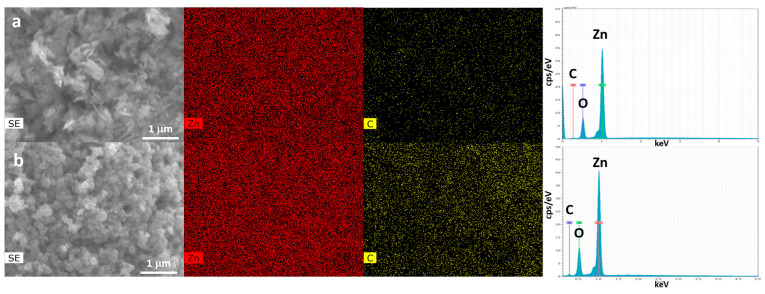
EDX elemental mapping of samples non-biogenic (**a**) and biogenic (**b**) ZnO-NPs.

The EDX elemental mapping of the samples (Figure 3) shows that the zinc distribution is homogeneous, and the ratio of Zn/O atomic% is c.a 1 for both samples. A much lower percentage of carbon is present on both sample surfaces (1.5 wt% for non-biogenic and 2 wt% for biogenic). These results are in line with XRD analysis.

### 3.3. Duckweed Phenolic Characterization

**Figure 4:**
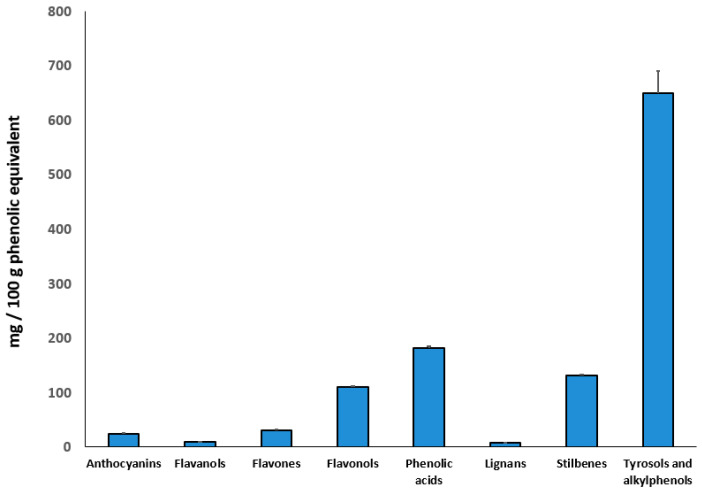
Abundance of phenolic compounds (mg 100 g^−1^) in the duckweed. Compounds have been screened by untargeted metabolomics, and cumulative abundance per each class quantified against a representative compound per each class.

According to an untargeted metabolomics approach, phenolic compounds were profiled in duckweed extracts. As provided in Figure 4, the extract was characterized by a diverse phenolic profile, where tyrosols, alkylphenols and other low molecular weight phenolics accounted for more than 50% of total phenolics (648.9 mg 100 g^−1^). The most represented compounds in this class were tyrosol and *p*-HPEA-AC, 4-hydroxycoumarin, the phenolic terpene carnosic acid and the alkylphenol 5-nonadecylresorcinol. Interestingly, the extract was also rich in flavonoids, with anthocyanins, flavonols, flavones, and flavonols being 24.5, 9.6, 31.2, and 110.1 mg 100 g^−1^ equivalents, respectively. In particular, the most abundant flavonoids were several glycosylated forms of kaempferol and quercetin, followed by peonidin and delphinidin derivatives, among others. Regarding phenolic acid contents (181.9 mg 100 g^−1^), the hydroxycinnamic, ferulic, caffeic, and coumaric acids were the most abundant. The whole list of phenolic compounds annotated in duckweed extracts is provided as Supplementary Materials, together with individual abundance, retention time, and composite mass spectra.

### 3.4. Effect of ZnO-NPs Synthesized Using Duckweed Extra on Maize Growth, Chlorophyll Content, Carotenoids, Anthocyanin and MDA

**Figure 5:**
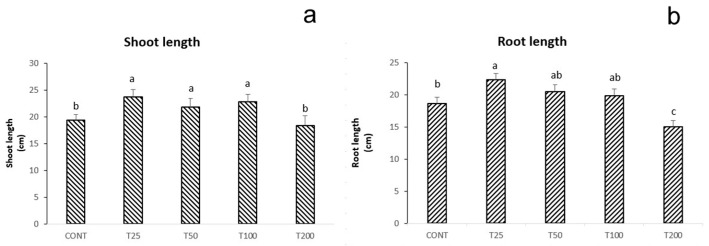
Shoot (**a**) and root (**b**) length recorded at 14 days after the treatments in maize seedlings subjected to the treatments with biogenic ZnO-NPs (T25, T50, T100 and T200) compared to the untreated control samples. Letters in the figure, when different, indicate statistically significant differences for *p* < 0.05 between treatments.

The concentration of the biogenic ZnO-NPs differently influenced the shoot and root length of maize seedlings at 14 DAS (Figure 5). Shoot length was positively affected by ZnO-NPs T25, T50, and T100, compared to control samples. Differently, the highest concentration T200 did not show significant differences with the untreated controls. As for root length, Figure 5b indicates that the only treatment capable of effectively increasing root length, if compared to control samples, was T25. All the other treatments did not show statistically significant differences from the control, except T200 that slightly decreased root length.

**Figure 6:**
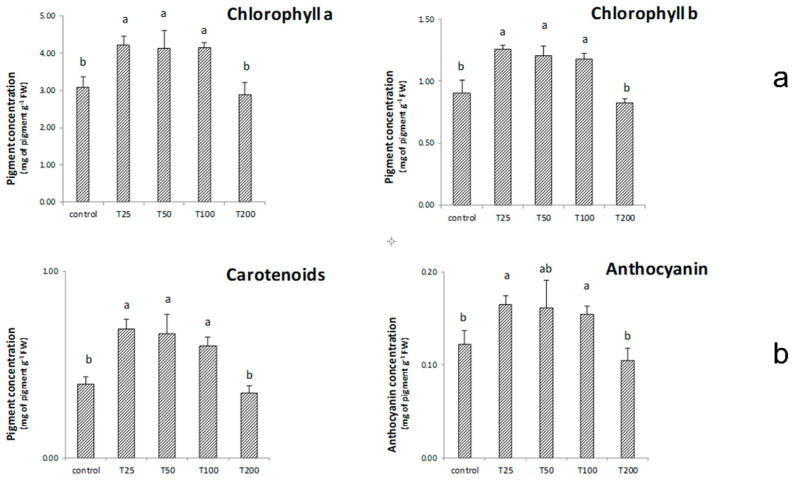
The figure shows the content of chlorophyll a and b (**a**) and on carotenoids and anthocyanin (**b**) recorded 14 days after the treatments with biogenic ZnO-NPs (T25, T50, T100 and T200) compared to the untreated control samples. Letters in the figure, when different, indicate statistically significant differences for *p* < 0.05 between treatments.

Figure 6a shows the effect of the different treatments on chlorophyll a and b in maize seedlings. Results indicated that T25, T50, and T100 significantly increased chlorophyll a and b concentration in treated samples than untreated controls. Further, the treatment T200 did not show any relevant effect.

As for carotenoids, T25, T50 and T100 determined substantial increases in their content. Moreover, T200 did not affect carotenoids (Figure 6b). Concerning anthocyanin, only the T25 and T100 showed values significantly higher than the controls (Figure 6b).

**Figure 7:**
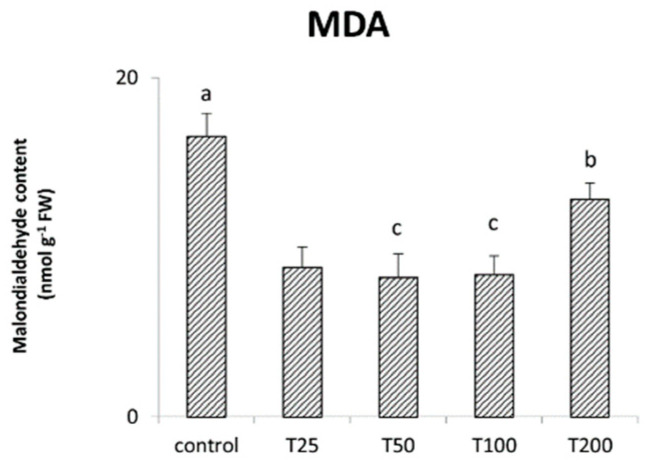
The figure shows the amount of MDA found 14 days after the treatments with biogenic ZnO-NPs (T25, T50, T100 and T200) compared to the untreated control samples. Letters in the figure, when different, indicate statistically significant differences for *p* < 0.05 between treatments.

The malondialdehyde content was determined in maize seedlings submitted to the different treatments with the biogenic ZnO-NPs (Figure 7). The highest concentration of MDA was found in the control samples. In contrast, all treatments with ZnO-NPs resulted in significant reductions in this index. In particular, T25, T50, and T100 showed the lowest values in MDA content.

## 4. Discussion

Nanotechnology can play a role in enhancing crop performance. Some research demonstrated that NPs could lead to highly positive and beneficial effects on crops, even if subjected to abiotic stress [4,7,22,39,40]. In this context, green synthesized ZnO-NPs show numerous advantages over the other conventional ones. In particular, green syntheses do not impact the environment, and biogenic ZnO-NPs, concerning their morphology, shape and size [22], can induce beneficial effects on treated crops [4,5,14].

In this context, a duckweed extract was used to carry out the biogenic synthesis of zinc oxide NPs. The NPs obtained applying the biogenic approach were then compared to those synthesized without the plant extract. In general, the XRD patterns showed by the ZnO-NPs indicated as both the biogenic and non-biogenic syntheses allowed obtaining nanometric structures (Figure 1). The biogenic synthesis effectiveness resulted in smaller NPs than those obtained applying the non-biogenic approach. In particular, the XRD patterns indicated dimensions of 11.7 nm for the biogenic NPs and 25.5 nm for the non-biogenic ones, respectively. Finally, the XRD analysis highlighted the absence of impurities for both synthetic approaches. The ZnO-NPs obtained using the duckweed extract showed a smaller average size than those reported in other studies [4,20,22]. It is well known that the size of NPs strongly depends on the chemical composition, classes, and amounts of metabolites contained in the biological entities [10,11,34].

The UV-vis spectra showed the typical absorption peak of ZnO-NPs in the range 370–380 nm (Figure 1). The maximum absorption depends on NPs morphology, dimension, and surface microstructure [18]. Biogenic NPs obtained in this study showed a slight blue shift (369 nm) compared to the non-biogenic ones (372 nm), and it can be attributable to the smaller dimensions and spherical shape of these NPs [41].

FE-SEM and TEM analyses indicated results in line with the XRD patterns (Figure 1 and Figure 2). The morphology of the biogenic NPs was quite different from that exhibited by non-biogenic ZnO-NPs. The biogenic ZnO-NPs showed a spherical structure, while the non-biogenic ones showed a lamellar structure. Finally, the non-biogenic ZnO-NPs tended to form aggregates up to 100 nm. Differently, the biogenic ZnO-NPs did not show appreciable aggregations. As for the shape of the biogenic ZnO-NPs, a wide variability can be observed depending on the biological entity used to carry out the biogenic syntheses. Such an effect is very frequently noted, and it mainly depends on the chemical composition of the plant extract [10,11]. For instance, some previous studies on plant biogenic synthesis evidenced as ZnO-NPs of flower or hexagonal shape can be obtained using *Carissa edulis*, *Justicia spicigera* and *Medicago sativa* [20,21,34]. In general, the possibility of controlling the shape of NPs is a crucial factor as it determines the properties of the realized nanomaterials [6,7,34].

As for the results of the untargeted metabolomic profile of the duckweed extract, the high number of phenolic compounds might have played a significant role in controlling the size and shape of the biogenic NPs (Figure 4). Indeed, scientific literature corroborated quercetin ability and other flavonoids in promoting the formation of nanosized materials displaying biological activity [34,42]. In particular, flavonoids show specific sites to bind metal ions, including hydroxyl groups [43].

**Figure 8:**
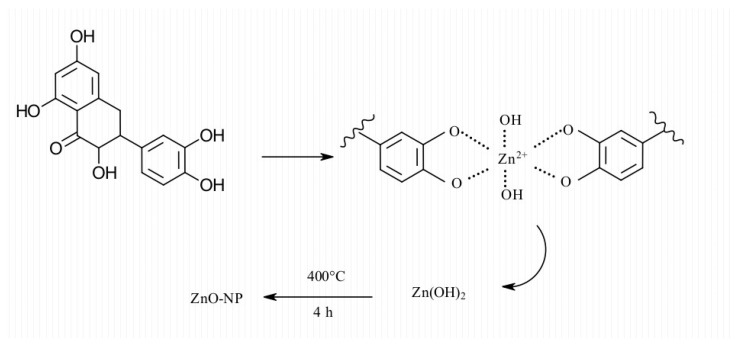
The proposed mechanism for ZnO-NPs formation.

No detailed mechanisms explain the effect of the phenolic moiety in NPs formation, but its contribution to reducing capacity and stabilization has been postulated [43]. The process may involve the relatively low oxygen-hydrogen bond dissociation energy of alcohol groups in the catechol moiety of flavonoids [44]. Phenolic antioxidants can reduce metal ions to NPs, thus providing stability against agglomeration [45,46]. A mechanism involving flavonoids (quercetin) is proposed in Figure 8.

Interestingly, it was reported that the specific plant source (and therefore, the associated phytochemical profile) could influence the characteristics of the NPs [34,47,48]. This implies that the choice of the plant source is pivotal in determining the features of biogenic NPs. This evidence allows us to speculate as the specific phenolic signature of duckweed extract may be responsible for our NPs characteristics. For the same reason, recent review articles suggest the importance of characterizing the nanomaterial synthesized [43,49].

Successively, the effect of the biogenic ZnO-NPs on maize was tested by direct application on seeds. In general, ZnO-NPs application to crops can prompt some beneficial effects for their ability to enhance defensive mechanisms and protect cell membrane structures, even though the mode of action of ZnO-NPs remains still unknown [7]. Seed-priming and foliar application of ZnO-NPs can improve plant performance [4]. On the contrary, NPs application at the root level can significantly decrease plant growth for the excessive Zn^2+^ released to roots from the NPs dissolution [7]. Such an effect is due to the alterations of root epidermis cells caused by the rapid dissolution of the ZnO-NPs, which hamper the treated species functionality [50]. Figure 5 illustrates the maize shoot and root growth following the treatments with the different ZnO-NPs concentrations. The dosages applied showed different effects. Shoot length was generally increased by ZnO-NPs, except for T200. This stimulatory effect on maize resulted from the ZnO-NPs induction of plant nutrition. Indeed, ZnO-NPs can upregulate the expression of genes involved in nutrient transporters in tomato plants [7].

In contrast, at the root level, the only effective treatment was T25, whereas T200 exerted an inhibiting effect. The T200 result can be explained based on the ZnO-NPs toxicity on crops resulting from excessive concentrations. Some studies documented the phytotoxic effects of NPs if applied in the range of 200 µg L^−1^ up to several thousand [51,52].

As for the content of chlorophyll a and b found in maize samples subjected to the various treatments, the intermediate doses induced significant increases in chlorophyll content compared to the control samples (Figure 6a). In contrast, the Chlorophyll a/b ratio was not affected by any applied NPs dosage (data not reported). The increase of photosynthetic pigments is a relevant aspect as it stimulates photosynthetic activity. In general, higher chlorophyll concentrations can also be considered a physiological adaptation to external stimuli, which enhance the plant capacity to harvest light in photosystems [53]. However, to explain the increases in chlorophyll, it is necessary to cite the zinc involvement in chlorophyll formation by protochlorophyllide and chloroplast development [54,55].

In contrast, T200, by providing excessive zinc to the crop, inhibited chlorophyll formation (Figure 6a). Such a result is the consequence of the interference on the expression of genes associated with chlorophyll biosynthesis, which can be observed at high ZnO-NPs concentrations [56]. This effect also explains the lower T200 effectiveness, compared to the other treatments, on plant biomass development.

Concerning carotenoids, ZnO-NPs showed some interesting effects (Figure 6b). The importance of carotenoids lies not only in their essential action in the photosynthetic process as light-harvesting pigments [57]. Indeed, these biomolecules have antioxidant activity and are involved in removing reactive oxygen species (ROS) [57]. Carotenoids protect chloroplasts from oxidative stress through their ability to quench chlorophyll in singlet or triplet form [58]. Our results indicate that T25, T50, and T100 resulted in significant increases in these biomolecules content. This result is in line with the effect of NPs, as they can lead to carotenoid production increases [39]. The carotenoid data, examined with the above, indicate that specific concentrations of ZnO-NPs can positively affect the pigment biosynthesis. Therefore, the use of non-excessive doses of ZnO-NPs can also be proposed as a valuable strategy to induce the content of these beneficial compounds [59]. On the other hand, the T200 data indicate that such concentrations interfered with carotenoids biosynthesis. This impairment has been previously reported in the literature following excessive concentrations of ZnO-NPs [60].

The biological role of anthocyanin is of pivotal importance as they can act against ROS in the vacuole, being considered antioxidants and inhibitors of lipid peroxidation [53,61]. In general, the anthocyanin content can increase in response to various environmental stresses [61]. Furthermore, increases in the anthocyanin biosynthesis have been proposed as a photo-protective response in rice leaves against photo-oxidative damages [53]. In this sense, the treatments with T25, T50, and T100 exerted an effect on anthocyanin content worthy of mention (Figure 6b). This induction can be intended as a photo-protective response of the treated maize to the ZnO-NPs. In fact, ZnO-NPs can promote photo-oxidative processes, thanks to the electronic structure of their valence electrons [62]. In particular, these electrons can be excited to the conduction band at wavelengths close to 400 nm [62]. Therefore, the plant perceived this signal at non-phytotoxic levels, thus increasing anthocyanin content. Furthermore, it can be stated as the increase in chlorophylls and anthocyanin can be linked. Indeed, it has been proposed that the rise in chlorophylls may be a possible physiological response that can compensate for eventual increases in anthocyanin [53]. This feedback mechanism is in agreement with our results.

Finally, the MDA content displayed by maize plants treated with the biogenic ZnO-NPs was ascertained (Figure 7). In general, all the treatments reduced the content of this lipid peroxidation product. MDA is mainly produced by oxidation of membranes of chloroplasts and mitochondria, organelles where metabolic processes with high electron flow occur [63]. When produced in high quantities, MDA is itself toxic to the cell. Increases in MDA content have been correlated with oxidative damage due to biotic and abiotic stresses [37,57]. In this context, our results indicate that zinc NPs led to a general reduction in MDA. The effects on MDA found for T25, T50, and T100 align with the increased content of carotenoids and anthocyanin recorded in this study. The beneficial effects of low NPs dosages on the oxidative state and MDA content have been ascertained in other studies, which showed as NPs application can result in ROS reduction [7,35,51].

On the contrary, this conclusion cannot be extended to T200. In this case, the reduction of MDA compared to control samples (Figure 7) may result from the activation of other protective and antioxidant mechanisms due to the severe impact of this treatment. This can be due to the onset of severe oxidative stress as it was found for maize treated with NPs at 800 µg L^−1^ [51] and soybean exposed to ZnO-NPs at 200 and 400 µg L^−1^ [64]. Likewise, T200 started to show some adverse effects on the various parameters studied compared to the other dosages.

**Figure 9:**
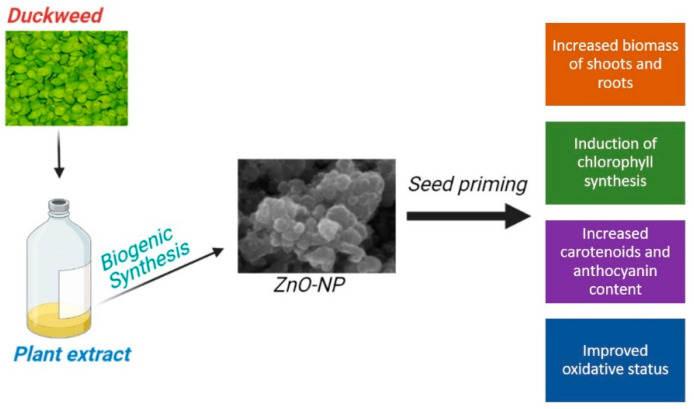
Summary of the positive effects of biogenic ZnO-NPs, obtained using duckweed extracts, in maize.

Therefore, when appropriate dosage and target uses have been defined, duckweed-based NPs synthesis can represent a promising approach for producing nanosized materials with potential biostimulant activity in crops (Figure 9). This ability aligns with the emerging applications of NPs in crop protection and agriculture [40,65].

## 5. Conclusions

This study has proven for the first time that by using an extract from duckweed, it is possible to obtain zinc oxide NPs. Then, we intend to highlight how unexplored natural resources available in nature can be exploited to synthesise valuable materials. Even in the context of green chemistry, the exploitation of such natural resources is a sustainable way forward as it has no environmental impact and is in line with the paradigm of the transition to a circular economy. Therefore, our research candidates the world of aquatic plants as an attractive environment to be better explored for finding biological entities that allow practical and sustainable synthesis of nanomaterials.

In detail, this research showed how duckweed could be successfully used to control ZnO-NPs, and this effect is evident when comparing the biogenic NPs with those synthesized without the plant extract. The biogenic ZnO-NPs were characterized by spherical shape and dimension of 11.7 nm (referred to as XRD determinations). Further, the plant-mediated synthesis showed satisfying efficiency in the ZnO-NPs synthesis of about 83.8%. The distinctive features of the duckweed were related to its high content of phenolic metabolites, capable of promoting efficient biogenic synthesis. Moreover, the untargeted metabolomics approach applied in this study, which is rarely used to screen the suitability of metabolites present in plant material for biogenic synthesis, has proved to be an advantageous technique for testing the adequacy of the biological entity for biogenic synthesis. Finally, it should also be emphasized as the approach developed in this work is noteworthy for being non-pathogenic and easily scalable, representing a valuable option when NPs are intended to be used for their biological activity.

Subsequently, maize seed priming revealed, at particular dosages, beneficial effects on development, pigments and antioxidants, which explain the low lipid peroxidation index. In light of the above, this research evidenced the possibility of using the obtained NPs to improve maize traits through seed nano-priming. This aspect may become strategically important considering the current context in which new substances have to be developed or used to increase crop yield and quality or increase their resistance to biotic and abiotic stresses. Indeed, the biostimulatory activity highlighted by the biogenic ZnO-NPs developed in this work is worthy of consideration.

Finally, given the value and numerous uses of the nanoparticle systems, a further possible application based on surface functionalisation should be considered. Biopolymeric systems can be obtained with metal oxide NPs functionalized with functional biopolymers [66]. This transformation leads to derivatives with improved properties suitable for biological and industrial uses [66].

## Data Availability

Data will be available on request to the corresponding.

## Supplementary Materials

The following are available online at https://www.mdpi.com/article/10.3390/nano11051270/s1, Table S1: Untargeted metabolomics of duckweed. The whole list of phenolic compounds annotated in duckweed extracts by untargeted metabolomics are reported in Table S1. Compounds are grouped in classes and are provided with individual abundances (n = 3) and composite mass spectra (monoisotopic mass—abundance combinations).
