# Supplementary material for: Biogenic ZnO Nanoparticles Synthesized Using a Novel Plant Extract: Application to Enhance Physiological and Biochemical Traits in Maize
Source: Nanomaterials (Basel). 2021 May 12;11(5):1270. doi: 10.3390/nano11051270 (PMC8151215; doi:10.3390/nano11051270)
Supplement: Supplementary file 1 [file nanomaterials-11-01270-s001.zip › nanomaterials-1194979-supplementary.pdf]

Supplementary material for Article

Biogenic ZnO Nanoparticles Synthesized Using a Novel Plant Extract: Application to Enhance Physiological and Biochemical Traits in Maize

Daniele Del Buono [1], Alessandro Di Michele [2], Ferdinando Costantino [3,*], Marco Trevisan [4] and Luigi Lucini [4]

[1] Dipartimento di Scienze Agrarie, Alimentari e Ambientali, University of Perugia, Borgo XX Giugno, 06121 Perugia, Italy; daniele.delbuono@unipg.it
[2] Department of Physics and Geology, University of Perugia, Via Elce di Sotto, 06123 Perugia, Italy; alessandro.dimichele@collaboratori.unipg.it
[3] Dipartimento di Chimica, Biologia e Biotecnologia, University of Perugia, Via Elce di Sotto 8, 06123 Perugia, Italy
[4] Department for Sustainable Food Process, Università Cattolica del Sacro Cuore, 29122 Piacenza, Italy; marco.trevisan@unicatt.it (M.T.); luigi.lucini@unicatt.it (L.L.)
* Correspondence: ferdinando.costantino@unipg.it; Tel.: +39-075-5855563

Table S1. Untargeted metabolomics of duckweed

Compound	class	subclass	repl. 1	repl. 2	repl. 3	Retention Time	CompositeSpectrum
Delphinidin 3-O-glucosyl-glucoside	Flavonoids	Anthocyanins	1348089	1300396	1287902	25.26	(612.1758, 7721.223)(613.17535, 1612.9067)
Cyanidin 3-O-glucosyl-rutinoside	Flavonoids	Anthocyanins	544990	523598	518408	26.69	(758.21356, 13293.754)(759.21405, 8281.646)(760.21234, 6216.8013)(761.2116, 1756.4116)
Delphinidin 3,5-O-diglucoside	Flavonoids	Anthocyanins	1348089	1300396	1287902	25.26	(612.1758, 7721.223)(613.17535, 1612.9067)
Cyanidin 3-O-sophoroside	Flavonoids	Anthocyanins	129509	112303	114434	15.32	(611.1779, 14230.013)(595.1574, 8364.227)(596.16034, 901.25665)(612.17596, 9758.246)(613.1755, 1982.7767)
Malvidin 3-O-(6''-acetyl-galactoside)/Malvidin 3-O-(6''-acetyl-glucoside)	Flavonoids	Anthocyanins	187221	181944	174395	24.46	(536.15845, 7875.565)(537.1589, 2269.1335)(538.15717, 315.0625)
Delphinidin 3-O-rutinoside	Flavonoids	Anthocyanins	129509	112303	114434	15.32	(611.1779, 14230.013)(595.1574, 8364.227)(596.16034,

								901.25665)(612.17596, 9758.246)(613.1755, 1982.7767)
Pigment A	Flavonoids	Anthocyanins	1348089	1300396	1287902	25.26	(610.1774, 23516.78)(611.1778, 11883.142)(612.1758, 7721.223)(613.17535, 1612.9067)
Cyanidin 3,5-O-diglucoside	Flavonoids	Anthocyanins	129509	112303	114434	15.32	(611.1779, 14230.013)(595.1574, 8364.227)(596.16034, 901.25665)(612.17596, 9758.246)(613.1755, 1982.7767)
Peonidin 3-O-rutinoside/Peonidin 3-O-(6''-p-coumaroyl-glucoside)	Flavonoids	Anthocyanins	1348089	1300396	1287902	25.26	(610.1774, 23516.78)(611.1778, 11883.142)(612.1758, 7721.223)(613.17535, 1612.9067)
Cyanidin 3-O-(6''-p-coumaroyl-glucoside)	Flavonoids	Anthocyanins	146252	122986	125393	5.38	(595.1574, 8364.227)(596.16034, 901.25665)
Delphinidin 3-O-(6''-p-coumaroyl-glucoside)	Flavonoids	Anthocyanins	129509	112303	114434	5.38	(595.1574, 8364.227)(596.16034, 901.25665)
Delphinidin 3-O-feruloyl-glucoside	Flavonoids	Anthocyanins	135157	120366	126427	5.61	(647.1483, 427.02)(625.16754, 9068.923)(626.17053, 1017.37665)
Petunidin 3-O-rutinoside/Pelargonidin 3-O-sophoroside	Flavonoids	Anthocyanins	146252	122986	125393	5.38	(595.1574, 8364.227)(596.16034, 901.25665)
Cyanidin 3-O-(6''-caffeoyl-glucoside)	Flavonoids	Anthocyanins	129509	112303	114434	5.38	(595.1574, 8364.227)(596.16034, 901.25665)
Cyanidin 3-O-rutinoside	Flavonoids	Anthocyanins	146252	122986	125393	5.38	(595.1574, 8364.227)(596.16034, 901.25665)
Petunidin 3,5-O-diglucoside	Flavonoids	Anthocyanins	135157	120366	126427	5.61	(625.1673, 9825.37)(626.1703, 953.67)(647.1483, 427.02)
Petunidin 3-O-(6''-p-coumaroyl-glucoside)	Flavonoids	Anthocyanins	142099	138302	138286	5.61	(625.16754, 9068.923)(626.17053, 1017.37665)
(+)-Catechin	Flavonoids	Flavanols	208873	195930	204294	7.11	(291.09396, 7694.814)(313.07535, 5652.69)
(+)-Gallocatechin	Flavonoids	Flavanols	202796	197678	187574	2.16	(306.07642, 9524.97)(307.07968, 993.25)(290.08243, 1844.1068)
Phloretin 2'-O-xylosyl-glucoside	Flavonoids	Dihydrochalcones	131587	137999	159235	7.46	(573.18695, 25149.297)(574.18945, 5575.607)

Neohesperidin/Hesperidin	Flavonoids	Flavanones	1623560	1570777	1542372	25.26	(610.1774, 24965.875)(611.1778, 12953.5205)
Neodiosmin/Diosmin	Flavonoids	Flavones	986179	945245	933800	25.26	(610.1774, 20799.129)(611.1778, 9922.528)(612.1758, 6447.187)(613.17535, 1347.225)
Nepetin	Flavonoids	Flavones	118697	116413	116717	1.39	(299.043, 9927.094)(300.04398, 3061.5266)
Tetramethylscutellarein	Flavonoids	Flavones	201826	195493	193794	5.95	(343.12228, 14359.937)(344.12476, 1315.7167)
Chrysoeriol 7-O-apiosyl-glucoside/Apigenin 6,8-di-C-glucoside/Luteolin 7-O-rutinoside	Flavonoids	Flavones	146252	122986	125393	5.38	(595.1574, 8364.227)(596.16034, 901.25665)
Kaempferol 3,7-O-diglucoside/Quercetin 3-O-galactoside 7-O-rhamnoside/Kaempferol 3-O-sophoroside/Quercetin 3-O-rutinoside	Flavonoids	Flavonols	1623560	1570777	1542372	25.26	(612.1758, 8434.604)(613.17535, 1762.6201)(611.1778, 12953.5205)
Kaempferol 3-O-glucosyl-rhamnosyl-glucoside	Flavonoids	Flavonols	379289	367652	362814	26.69	(758.21356, 12976.849)(759.21405, 7940.65)(760.21234, 5961.029)(761.2116, 1684.1034)
Quercetin 3-O-rhamnosyl-galactoside	Flavonoids	Flavonols	1623560	1570777	1542372	25.26	(612.1758, 8434.604)(613.17535, 1762.6201)(611.1778, 12953.5205)
Kaempferol 3-O-glucosyl-rhamnosyl-galactoside	Flavonoids	Flavonols	379289	367652	362814	26.69	(758.21356, 12976.849)(759.21405, 7940.65)(760.21234, 5961.029)(761.2116, 1684.1034)
Quercetin 3-O-rhamnosyl-rhamnosyl-glucoside	Flavonoids	Flavonols	379289	367652	362814	26.69	(758.21356, 12976.849)(759.21405, 7940.65)(760.21234, 5961.029)(761.2116, 1684.1034)
6,8-Dihydroxykaempferol	Flavonoids	Flavonols	2790476	2724843	2726585	5.98	(341.01947, 616.77)(319.03937, 149745.45)(320.0419, 16677.469)(321.04034, 508.075)
Isorhamnetin	Flavonoids	Flavonols	118697	116413	116717	1.39	(299.043, 9927.094)(300.04398, 3061.5266)
Kaempferol 3-O-rutinoside	Flavonoids	Flavonols	146252	122986	125393	5.38	(595.1574, 8364.227)(596.16034, 901.25665)

Myricetin	Flavonoids	Flavonols	2790476	2724843	2726585	5.98	(341.01947, 616.77)(319.03937, 149745.45)(320.0419, 16677.469)(321.04034, 508.075)
Quercetin 3-O-xylosyl-glucuronide	Flavonoids	Flavonols	746335	732066	737320	5.98	(615.08716, 42211.4)(616.08905, 9811.126)(617.0774, 2988.01)
Rhamnetin	Flavonoids	Flavonols	118697	116413	116717	1.39	(299.043, 9927.094)(300.04398, 3061.5266)
Isorhamnetin 3-O-glucoside 7-O-rhamnoside	Flavonoids	Flavonols	147729	138302	138286	5.61	(647.1483, 427.02)(625.1673, 9825.37)(626.1703, 953.67)
Isoferulic acid/Ferulic acid	Phenolic acids	Hydroxycinnamic acids	2145806	2098528	2089305	7.36	(177.05347, 83174.09)(178.05617, 6248.8696)(201.04121, 10838.944)(203.0504, 2057.28)
p-Coumaroyl malic acid	Phenolic acids	Hydroxycinnamic acids	119268	115476	115010	6.95	(281.06625, 1359.5367)(303.0432, 5027.4634)
Caffeic acid	Phenolic acids	Hydroxycinnamic acids	5623987	5467105	5419568	5.98	(185.06374, 8213.83)(163.03793, 220429.38)(164.0407, 16307.753)
o-Coumaric acid/p-Coumaric acid/m-Coumaric acid	Phenolic acids	Hydroxycinnamic acids	280101	302421	286450	2.13	(165.05305, 16083.914)(168.06136, 3322.867)(147.0419, 2958.5967)
Cinnamic acid	Phenolic acids	Hydroxycinnamic acids	189788	188061	194672	2.73	(149.0581, 6252.15)(131.04767, 5045.14)
p-Coumaroyl tartaric acid	Phenolic acids	Hydroxycinnamic acids	2774366	2724843	2721429	5.98	(319.0394, 149131.64)(320.0419, 16572.127)(321.04028, 511.19333)
Sinapine	Phenolic acids	Hydroxycinnamic acids	807651	800273	778141	3.41	(333.1481, 40457.066)(334.15088, 3920.0203)
Homoveratric acid	Phenolic acids	Hydroxyphenylacetic acids	153636	148461	154157	7.36	(204.06067, 2738.8633)(205.06624, 5933.5034)
4-Hydroxyphenylacetic acid	Phenolic acids	Hydroxyphenylacetic acids	189723	185864	187687	2.16	(137.04506, 3735.11)(153.05693, 8292.696)
Homovanillic acid/Dihydrocaffeic acid	Phenolic acids	Hydroxyphenylacetic acids	237124	253769	243069	2.13	(185.06384, 9106.32)(165.05305, 14771.997)
Anhydro-secoisolariciresinol	Lignans	Lignans	165301	164208	167079	6.162	(367.15463, 9237.667)(349.14453, 2421.6833)
Resveratrol 3-O-glucoside/Resveratrol 5-O-glucoside	Stilbenes	Stilbenes	670746	662292	649620	6.162	(390.14014, 32490.709)(391.13205, 5498.9766)

Piceatannol 3-O-glucoside	Stilbenes	Stilbenes	4466405	4474697	4406861	6.162	(391.13205, 5498.9766)(411.11725, 3089.9)
p-HPEA-AC	Other polyphenols	Tyrosols	1905260	325222	2430921	1.8623334	(182.07956, 11061.445)(183.0824, 799.325)(164.07236, 9954.865)(165.0757, 515.06)(166.0853, 45848.22)(167.0879, 3519.1602)(206.0789, 61.92)(186.73332, 15367.487)(187.46553, 11754.587)(188.0674, 886.59)
3,4-DHPEA-EDA	Other polyphenols	Tyrosols	201826	195493	198987	5.945	(343.12228, 14000.55)(344.12476, 1283.2034)
3,4-DHPEA-AC	Other polyphenols	Tyrosols	153636	148461	154157	7.3629994	(204.06067, 2738.8633)(205.06624, 5933.5034)
Hydroxytyrosol	Other polyphenols	Tyrosols	2124202	2087590	2084042	7.3629994	(177.05347, 83174.09)(178.05617, 6248.8696)
Bisdemethoxycurcumin	Other polyphenols	Curcuminoids	208873	195930	204294	7.1129994	(291.09396, 7694.814)(313.07535, 5652.69)
p-Anisaldehyde	Other polyphenols	Hydroxybenzaldehydes	260664	260261	253899	2.125	(119.047264, 2669.4768)(123.05404, 40229.035)(137.0617, 7518.096)
Vanillin	Other polyphenols	Hydroxybenzaldehydes	189723	185864	187687	2.1590002	(137.04506, 3735.11)(153.05693, 8292.696)
Syringaldehyde	Other polyphenols	Hydroxybenzaldehydes	237124	253769	243069	2.125	(185.06384, 9106.32)(165.05305, 14771.997)
Ferulaldehyde	Other polyphenols	Hydroxycinnamaldehydes	153636	148461	154157	7.3629994	(204.06067, 2738.8633)(205.06624, 5933.5034)
4-Hydroxycoumarin/Umbelliferone	Other polyphenols	Hydroxycoumarins	5654984	5498468	5457679	3.6995	(163.03793, 220429.38)(164.0407, 16307.753)(145.02684, 1476.3766)(168.04893, 5177.217)(167.01064, 5883.1704)(168.0107, 874.29663)
Mellein	Other polyphenols	Hydroxycoumarins	153636	148461	154157	7.3629994	(204.06067, 2738.8633)(205.06624, 5933.5034)

Guaiacol	Other polyphenols	Methoxyphenols	122458	120462	121144	2.7180002	(107.0439, 12339.33)(152.0552, 4312.705)
Thymol	Other polyphenols	Phenolic terpenes	347013	282785	317232	2.076	(133.104, 17294.5)(177.1002, 2758.5234)
Carnosic acid	Other polyphenols	Phenolic terpenes	2716898	2592367	2646366	13.584	(333.19986, 45602.594)(334.20267, 6650.31)(335.2138, 1066.27)
Carnosol	Other polyphenols	Phenolic terpenes	323351	320697	315232	15.352	(331.1827, 12112.144)(315.18918, 30034.904)(316.19238, 3266.6335)
Rosmadial	Other polyphenols	Phenolic terpenes	165301	164208	167079	6.162	(367.15463, 9237.667)(349.14453, 2421.6833)
Carvacrol	Other polyphenols	Phenolic terpenes	347013	282785	317232	2.076	(133.104, 17294.5)(177.1002, 2758.5234)
4-Vinylsyringol	Other polyphenols	Alkylmethoxyphenols	231540	220560	218686	2.643	(242.09727, 4951.7397)(265.07642, 6452.754)
5-Nonadecenylresorcinol	Other polyphenols	Alkylphenols	1383718	1365768	1379982	8.044999	(397.29868, 68142.18)(398.30045, 1702.64)
3-Methylcatechol	Other polyphenols	Alkylphenols	122458	120462	123255	2.7180002	(107.0439, 12339.33)(152.0552, 4312.705)
4-Vinylphenol	Other polyphenols	Alkylphenols	671772	656575	660965	2.726	(103.0534, 30908.695)(104.056656, 248.07)
